# Defining the kinetic effects of infection with influenza virus A/PR8/34 (H1N1) on sphingosine-1-phosphate signaling in mice by targeted LC/MS

**DOI:** 10.1038/s41598-021-99765-0

**Published:** 2021-10-11

**Authors:** Divyavani Gowda, Marumi Ohno, Siddabasave Gowda B. Gowda, Hitoshi Chiba, Masashi Shingai, Hiroshi Kida, Shu-Ping Hui

**Affiliations:** 1 Faculty of Health Sciences, Hokkaido University, Kita-12 Nishi-5, Kita-Ku, Sapporo, 060-0812 Japan; 2 Department of Nutrition, Sapporo University of Health Sciences, Nakanuma Nishi-4-3-1-15, Higashi-Ku, Sapporo, 007-0894 Japan; 3 International Institute for Zoonosis Control, Hokkaido University, Kita 20 Nishi10, Kita-ku, Sapporo, 001-0020 Japan

**Keywords:** Biochemistry, Microbiology, Biomarkers, Diseases, Chemistry

## Abstract

Influenza remains a world-wide health concern, causing 290,000–600,000 deaths and up to 5 million cases of severe illnesses annually. Noticing the host factors that control biological responses, such as inflammatory cytokine secretion, to influenza virus infection is important for the development of novel drugs. Sphingosine-1-phosphate (S1P) is a bioactive sphingolipid metabolite and has essential biological functions in inflammation. However, the kinetic effects of influenza virus infection on physiological S1P levels and their signaling in multiple tissues remain unknown. In this study, we utilized a mouse model intranasally infected with 50 or 500 plaque forming units (PFU) of A/Puerto Rico/8/34 (H1N1; PR8) virus to investigate how S1P levels and expression of its regulating factors are affected by influenza virus infection by the liquid-chromatography/mass spectrometry and real-time PCR, respectively. The S1P level was significantly high in the plasma of mice infected with 500 PFU of the virus than that in control mice at 6 day-post-infection (dpi). Elevated gene expression of sphingosine kinase-1 (*Sphk1*), an S1P synthase, was observed in the liver, lung, white adipose tissue, heart, and aorta of infected mice. This could be responsible for the increased plasma S1P levels as well as the decrease in the hepatic S1P lyase (*Sgpl1*) gene in the infected mice. These results indicate modulation of S1P-signaling by influenza virus infection. Since S1P regulates inflammation and leukocyte migration, it must be worth trying to target this signaling to control influenza-associated symptoms.

## Introduction

Influenza, a respiratory disease caused by an influenza virus infection, continues to threaten humans and remains a worldwide health concern. Although influenza is a disease of host immune response to the virus infection, particularly excess cytokine secretion, energy and fatty acid metabolism disorders have been suggested to be associated with the disease severity by previous studies representing higher mortality in patients or experimental animals with obesity, diabetes, and abnormal fatty acid oxidation^1–8^. Recently, sphingolipids, particularly sphingosine-1-phosphate (S1P), have emerged as molecules that modulate symptoms through their functions, e.g., the preservation of the integrity of the vascular endothelium and suppression of excessive cytokine responses, during influenza, dengue, and coronavirus disease 2019^9–15^. Therefore, S1P agonism is considered to protect the host against influenza and other infectious diseases. This notion is supported by a previous study that S1P antagonists exacerbated cytokine storm and lethality during influenza in mice^16^.

Sphingolipids, including sphingosine, ceramide, sphingomyelin, and S1P, are structural components in the plasma membranes of eukaryotic cells, characterized by hydrocarbon backbones having 2-amino-1,3-diol-termed sphingoid bases^17^. S1P is synthesized by sphingosine kinase (SPHK) isoenzymes SPHK1 and SPHK2, and decomposed into hexadecenal and phosphoethanolamine by the action of S1P lyase 1 (SGPL1) or converted to sphingosine by S1P phosphohydrolases as a part of sphingolipid rheostat^18,19^. S1P synthesized inside the cell is extracellularly transported via multiple S1P transporters and wields pleiotropic effects through binding to G protein-coupled S1P receptors (S1PRs)^20^. S1P is present at high concentrations in the blood by the contribution of platelets^21^, red blood cells^22^, and endothelial cells^23^, but at low levels in tissues, and this concentration gradient is critical for S1P to exert its roles as a signaling molecule in preserving vascular endothelial cell barrier integrity and regulating the migration of immune cells through S1PRs^24,25^. As described earlier, S1P signaling is thought to be involved in the severity of infectious diseases, but S1P signaling is also modified in many viral infections through changes in the expression and activity of these enzymes and transporters, especially SPHK1^26^. Since viral contact with host cell membranes occurs during viral entry and budding, it is not surprising that the sphingolipid metabolic system, a major component of cell membranes, is affected by a viral infection, as previously reviewed^27^. Given a possible protective role of S1P signaling in viral infectious diseases, it is important to understand how S1P metabolism is affected during viral infections. However, most of these findings have been obtained from experiments in cultured cells, and there are only a few reports of longitudinal observations of S1P concentrations during viral infection in vivo.

Here, we investigated the kinetic profile of S1P levels in the plasma and liver with liquid chromatography/mass spectrometry (LC/MS) utilizing an influenza mouse model which had been established previously^1^. In addition, we analyzed gene expression levels of metabolic enzymes, receptors, and cellular transporters of S1P to explore possible regulating mechanisms of the lipid in response to influenza virus infection.

## Materials and methods

### Materials

The LC/MS grade solvents methanol, acetonitrile, and isopropanol were obtained from Kanto Chemical Co. Ltd (Tokyo, Japan). The mobile phase additives acetic acid, ammonium acetate, and extraction solvent chloroform of LC/MS grade were obtained from Sigma-Aldrich (St. Louis, MO, USA). Ceramide/Sphingoid internal standard mixture II and sphingosine-1-phosphate were obtained from Avanti Polar Lipids (Alabaster, AL, USA). Sphingosine was obtained from Funakoshi Frontiers in Life Science (Tokyo, Japan). Phosphate-buffered saline (PBS) was purchased from Gibco/Life Technologies (Carlsbad, CA, USA).

### Virus

Influenza virus A/Puerto Rico/8/34 (H1N1; PR8) was kindly provided by the National Institute of Infectious Diseases in Japan. The virus was propagated in 10-day-old embryonated chicken eggs at 35 °C for 48 h, and aliquots of collected allantoic fluids were stored at − 80 °C.

### Mice

As reported previously^1^, male C57BL/6 mice were purchased from Hokudo (Sapporo, Japan) and were kept at a BSL-2 laboratory at the International Institute for Zoonosis Control, Hokkaido University, under standard laboratory conditions (room temperature 22 °C ± 2 °C, relative humidity 50% ± 10%) and a 12/12-h light/dark cycle^1^. The mice were administered a standard CE-2 chow diet purchased from CLEA Japan (Sapporo, Japan) with water ad libtum. Experiments were performed on 11–12 week-old mice. All the animal protocols were approved by the Animal Care and Use Committee of Hokkaido University (approval no. 17-0003).

### Virus infection and sample collection

**Figure 1:**
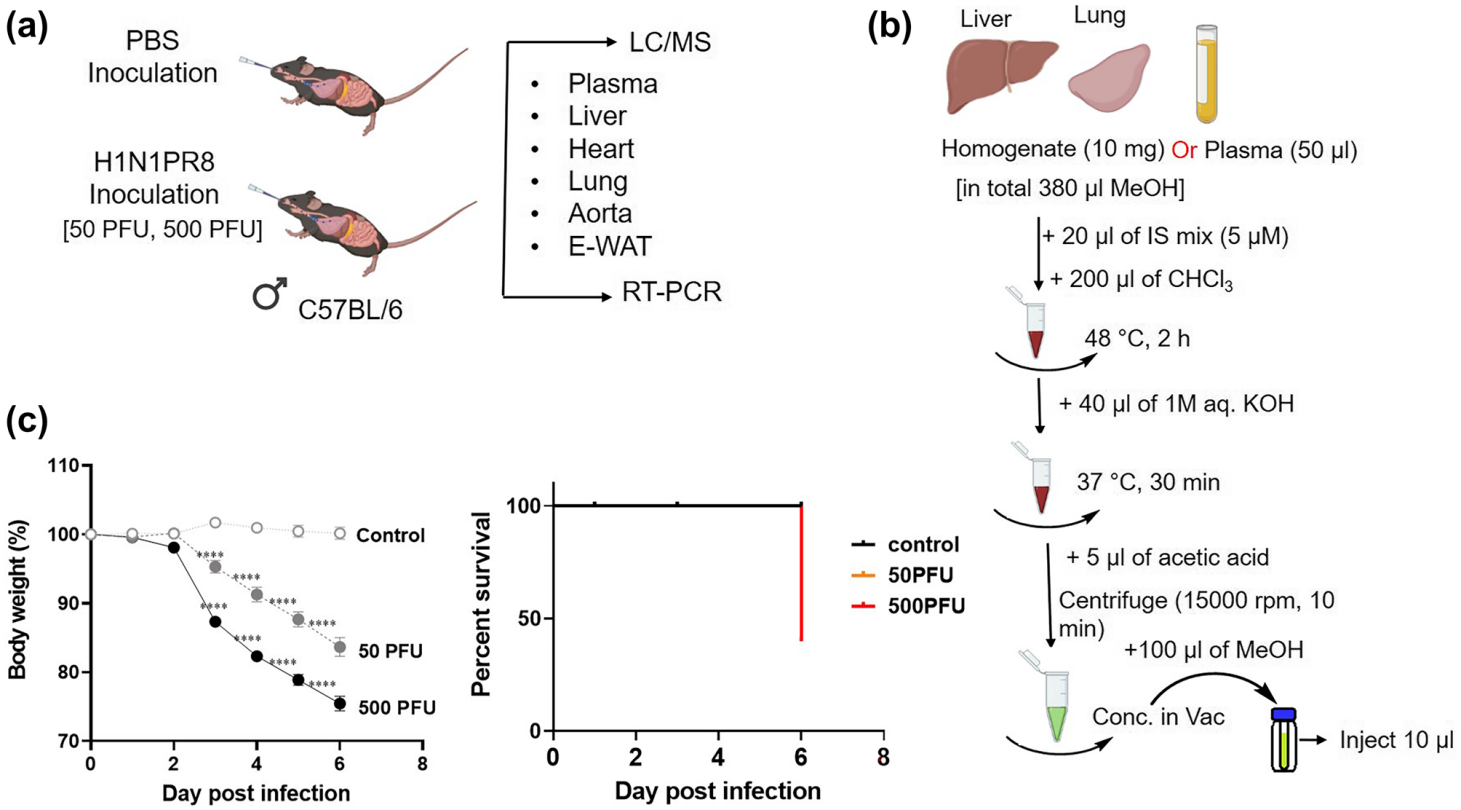
Experimental design (**a**), sphingolipid extraction workflow (**b**), and changes in body weight and survival rate (**c**), after virus inoculation. Mice were intranasally inoculated with PBS control or PBS comprising PR8 virus (50 or 500 PFU), and body weight was daily monitored until 6 dpi. A total of 15 mice/group were used for the experiment and 5 mice each were euthanized for sample collection on 1, 3, and 6 dpi. The mean body weight of mice is represented as a percentage of the original weight (**c**). White, gray, and black symbols indicate PBS control, 50 PFU, and 500 PFU PR8 virus infected mice, respectively; ****p < 0.0001, Mixed-effects analysis with Tukey’s multiple comparison test. (**c**) Percent survival of each group is shown. Black, orange, and red bars indicate PBS control, 50 PFU, and 500 PFU PR8 virus infected mice, respectively. Survival curves are compared using the log-rank (Mantel–Cox) analysis; *p < 0.05. Images are adopted from licensed BioRender.com.

PR8 virus at 50 or 500 plaque-forming units (PFU) in 50 µL of PBS or PBS only (control) were intranasally inoculated into the mice under inhalation anaesthesia with isoflurane (Fig. 1a). Body weight losses were monitored daily after infection, and mice were humanely euthanized when weight loss reached 25%. On 1, 3, or 6 days-post-infection (dpi), the mice were euthanized, and their liver and blood samples were collected. Blood samples were centrifuged at 1000*g* for 20 min at 4 °C in the presence of sodium heparin, and supernatants were collected as plasma. Each plasma sample was mixed with 4 volumes of methanol. Each liver sample was weighed and minced in 10 volumes of methanol. Plasma and liver samples with methanol were stored at − 80 °C until further lipid analyses.

### Measurement of selected gene expressions using real-time PCR

For gene expression analyses, PR8 virus at 500 PFU in 50 µL in PBS or PBS only (control) were intranasally inoculated into the mice under inhalation anaesthesia with isoflurane. On 1, 3, or 6 dpi, the mice were euthanized, and their liver, epididymal white adipose tissue (e-WAT), heart, lung, and thoracic aorta samples were collected and homogenized in 200–500 μL of TRIzol (Thermo Fisher Scientific, Waltham, MA, USA) and stored at − 30 °C until RNA extraction. Total RNA was extracted from tissue samples homogenized in TRIzol (Thermo Fisher Scientific) and used for cDNA synthesis using High-Capacity cDNA Reverse Transcription Kits (Thermo Fisher Scientific), according to the manufacturer’s instruction. The gene expressions of sphingosine kinase 1 (*Sphk1*, Mm00448841_g1), sphingosine phosphate lyase 1 (*Sgpl1*, Mm00486079_m1), sphingosine-1-phosphate receptor 1 (*S1pr1*, Mm02619656_s1), *S1pr3* (Mm02620181_s1), ATP-binding cassette, sub-family A (ABC1), member 1 (*Abca1*, Mm00442646_m1), ATP-binding cassette, sub-family G member 2 (Junior blood group) (*Abcg2*, Mm00496364_m1), ATP-binding cassette, sub-family C (CFTR/MRP), member 1 (*Abcc1*, Mm00456156_m1), and spinster homolog 2 (*Spns2*, Mm01249324_m1) were quantified with commercially available TaqMan probes (Applied Biosystems, Foster City, CA). In addition, the following specific primers and a probe were used for the measurement of *S1pr2* gene expression: forward primer, 5′-AGACGCCAAGGCCACTCA-3′; reverse primer, 5′-TTCCAGAACCTTCTCAGGATTGA-3′; probe (FAM), 5′-CTAGCCAGTGCTCAGC-3′. The concentrations of primers and probes in the reaction were 900 nM and 250 nM, respectively. The real-time PCR reaction was performed with a Step One Real-Time PCR system (Applied Biosystems). The obtained gene expressions were normalized to those of *18S* (Mm03928990_g1) from the same samples, and relative expressions were calculated using the comparative Ct method. The cut-off value of the Ct value used in the analysis was set to 35. The ddCt values of all the analyzed genes in this study were provided in Supplementary Table S1.

### Extraction of S1P

S1P was extracted from the liver, lung, and plasma by the method established earlier with minor modifications^28^. In brief, liver or lung tissue weighed was transferred into a 1.5 mL Eppendorf tube, and methanol (10 µL/mg) was added to normalize the weight and deactivate the virus. Five to six ceramic beads (1.4 mm, catalog no. 15-340-159, Fisher brand, Waltham, MA) were added to the tubes, and the tissue was homogenized (30 s × 2) using Bead Mill 4 (Fisher brand) homogenizer. About 100 µL (10 mg) of the liver homogenate or 50 µL of plasma was used for the extraction of S1P. Cer/Sph mixture II (Avanthi Polar Lipids), diluted to 5 µM with methanol, was used as an internal standard (IS mix). The detailed workflow of the extraction was described in Fig. 1b. The amount of sample injected into LC/MS was 10 µL.

### LC/MS analysis

The single reaction monitoring (SRM) analysis was performed using a Surveyor HPLC system coupled to a TSQ Quantum 124 Access MAX mass spectrometer with a heated electrospray ionization (H-ESI) probe (Thermo Fisher Scientific). Chromatographic separation was achieved using Hypersil gold C8 column (5 cm × 2.1 mm I.D., 5 µm, Thermo Fisher Scientific) maintained at 40 °C with a flow rate of 300 µL/min. The mobile phase consisted of A:10 mM ammonium acetate with 500 nM EDTA and 0.1% acetic acid, B: acetonitrile, C: isopropanol, and D: methanol. The elution gradient was: 80% A, 10% B, and 10% D (up to1 min); 50% A, 10% B, 30% C, and 10% D (5–11 min), 10% A, 20% B, 60% C, and 10% D (11–15 min), 5% A, 95% C (15.1–18 min). The SRM under positive mode was utilized for MS detection, and the optimized parameters were as follows: Spray voltage (4000 V), vaporizer temperature (250 °C), capillary temperature (300 °C), and nitrogen was used as the sheath gas and the auxiliary gas with a flow of 40 psi and 55 psi, respectively. Argon was used as the collision gas. Peak alignment, peak integration with smoothing points 7, area noise factor 5, peak noise factor 10, and quantification were performed using Thermo Xcalibur 2.1 software (Thermo Fisher Scientific). The total ceramide, hexosylceramide, and sphingomyelin relative levels were determined by the untargeted analysis method established earlier in our laboratory using LTQ-Orbitrap-MS in negative ionization mode^29^. The details of total lipid extraction, lipid annotation, and relative quantification were provided in supporting information.

### Statistical analysis

Two-way ANOVA with Tukey or Sidak multiple comparison tests was applied using GraphPad prism 8.0.1 software with a GP value of 0.1234 (ns), 0.0332 (*), 0.0021 (**), 0.0002(***), 0.0001(****). All the data were represented as the mean ± standard error.

### Ethical statement

All mouse experiments were performed in accordance with ARRIVE guidelines (https://arriveguidelines.org) with prior approval from the Animal Care and Use Committee of Hokkaido University. Further, following the Fundamental Guidelines for Proper Conduct of Animal Experiment and Related Activities in Academic Research Institutions under the jurisdiction of the Ministry of Education, Culture, Sports, Science and Technology in Japan. Body weight losses were monitored daily after infection, and mice were humanely euthanized when weight loss reached 25%.

## Results

### Change in body weight of mice upon PR8 virus infection

Mice were intranasally infected with PR8 virus at a dose of 50 or 500 PFU for models of mild or severe influenza, respectively. Body weight changes were monitored daily until 6 dpi, as shown in Fig. 1c. While control mice had almost unchanged body weights during the observation period, the infected mice in both groups (50 and 500 PFU) showed a significant decrease in the body weight starting at 3 dpi. Body weight loss in mice infected with 50 and 500 PFU of PR8 virus was almost 4% and 13% at 3 dpi, and 17% and 25% at 6 dpi, respectively. In the severe influenza model group (500 PFU), 3 of 5 mice met the criteria for euthanasia because their weight loss reached a humane endpoint (25 %) at 6 dpi. Thus, the survival rate in the group was dropped to 40% at that time point (Fig. 1c). To investigate the effect of influenza virus infection on S1P signalling, plasma and tissues were collected at 1, 3, and 6 dpi for samples at very early-stage, the onset of symptoms, and the lethal phase during influenza, respectively.

### Alteration of S1P quantity in the liver, lung, and plasma of mice infected with PR8 virus

**Figure 2:**
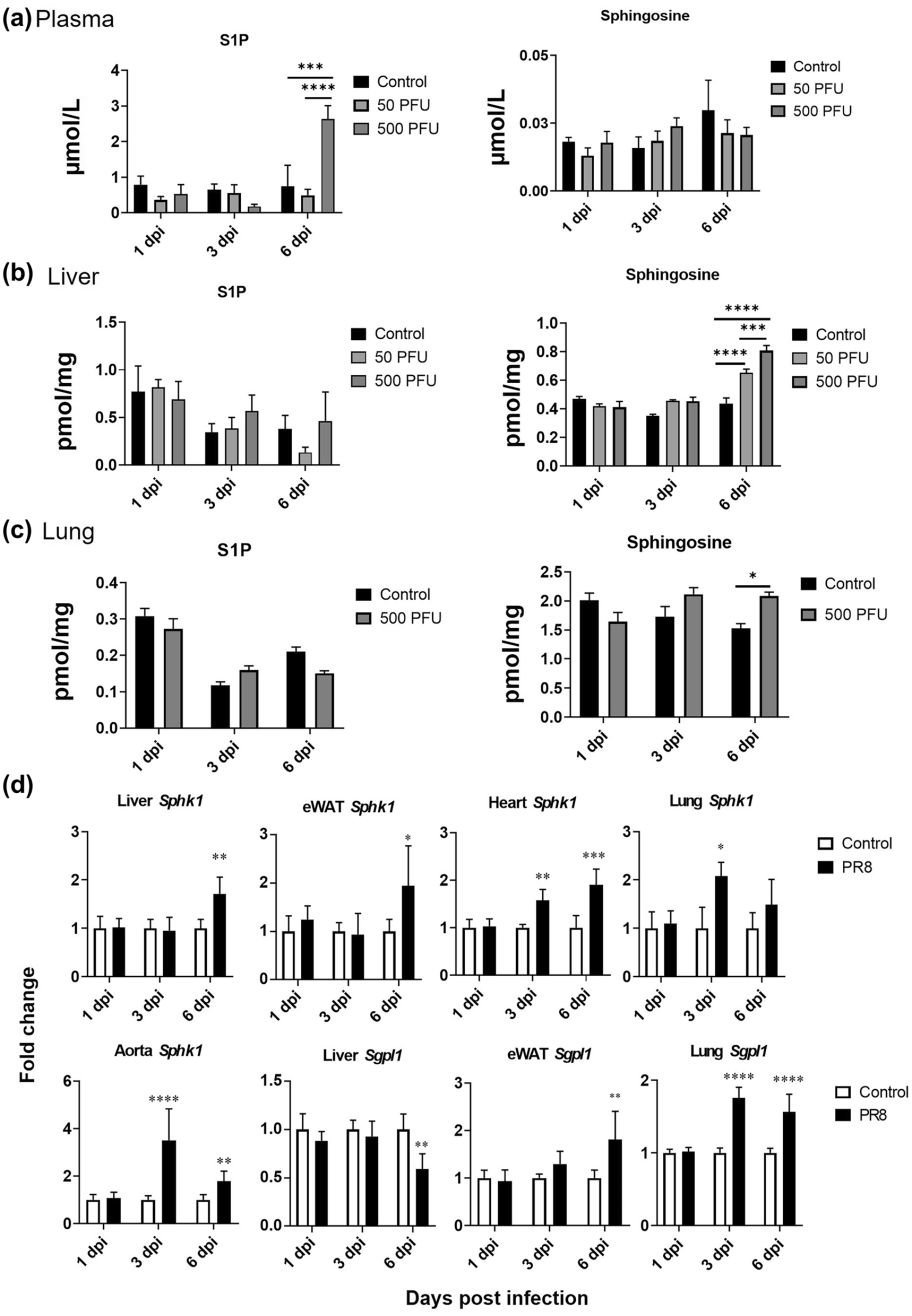
Concentrations of sphingolipids, gene expressions of S1P synthesis (*Sphk1*), and hydrolysis enzymes (*Sgpl1*) Concentrations of S1P and its precursor sphingosine in plasma (**a**), liver (**b**), and lung (**c**) of PR8 virus infected mice (n = 5 each, mean ± SE, Two-way ANOVA with Tukey’s multiple comparison test). Mice were intranasally inoculated with PBS alone or PBS comprising PR8 virus (500 PFU), and liver, e-WAT, lung, heart, and aorta samples were collected at 1, 3, and 6 dpi. Expressions of target genes (**d**) normalized with that of 18S are presented as fold changes relative to those of the control mice at each time point. Bars represent means ± SEM of 7 or 8 (liver, lung, aorta) or 3 or 4 (e-WAT, heart) animals. White and black bars indicate data from control and PR8 virus-infected mice, respectively; *p < 0.05, **p < 0.005, ***p < 0.0005, 2way-ANOVA on ddCt, control vs. PR8 virus-infected mice at each time point.

The quantitative analysis of targeted sphingolipids such as S1P and its precursor sphingosine in the liver and plasma of mice infected with PR8 virus was performed by LC/MS, and their extracted ion chromatograms are shown in Supplementary Fig. S1. The S1P levels ranged from 0.19 to 2.64 µmol/L in the plasma and 0.13 to 0.82 pmol/mg in the liver. Sphingosine levels in the plasma and liver ranged from 0.01 to 0.03 µmol/L and 0.34 to 0.80 pmol/mg, respectively. The acquired plasma concentrations of S1P are almost consistent with the previous reports, which showed a wide variance from 0.1 to 0.8 µmol/L^30^ and 1 to 3 µmol/L^14^ in mice. In the plasma, the sphingosine levels were unaltered on days 1, 3, and 6 after infection. However, compared to controls, plasma S1P levels were significantly increased by about fivefold at 6 dpi in mice infected with PR8 virus at 500 PFU (Fig. 2a). Conversely, S1P levels in the liver were not significantly altered in both infection groups during the experimental period, and the liver sphingosine content increased 1.5- to 2-fold in mice infected with 50 or 500 PFU of PR8 virus at 6 dpi, compared to controls (Fig. 2b). Additionally, in the lung of infected mice, the levels of S1P were not changed, but a significant increase in those of sphingosine was observed (Fig. 2c). Sphingolipids are known to be involved in multiple signal transduction pathways to exert their diverse biological functions. Hence, the mechanisms under which S1P and sphingosine levels were altered during virus infection need to be further investigated.

### Alteration of gene expressions of S1P synthase and hydrolases in mice infected with PR8 virus

The gene expression levels of S1P synthases (*Sphk1*, *Sphk2*) and S1P hydrolases (*Sgpl1*) in the liver, e-WAT, lung, heart, and aorta of mice were measured by real-time PCR under the infection conditions where a significant increase in plasma S1P concentration was observed. In mice infected with PR8 virus at 500 PFU, the gene expression levels of *Sphk1* significantly increased by 1.5- to 3.5-fold in the lung, heart, and aorta at 3 dpi and twofold in the liver, e-WAT, heart, and aorta at 6 dpi, compared to controls at each time point (Fig. 2d). However, no apparent changes were observed in *Sphk2* levels in all tested tissues (Supplementary Fig. S2). Furthermore, a slight reduction in *Sgpl1* levels was observed in the liver of the PR8 virus infected mouse at 6 dpi. In contrast, the *Sgpl1* level in lung and e-WAT increased by twofold. Increased expression levels of *Sphk1* in multiple tissues indicated the activation of S1P synthesis during influenza. Apart from this, decreased *Sgpl1* observed in the liver of infected mice at 6 dpi may have contributed to the suppression of S1P degradation in the tissue; however, the hepatic S1P level was unchanged. Hence, further mechanistic investigations are necessary to elucidate the effect of influenza virus infection on sphingolipid metabolism in the liver.

### Alteration of gene expression of S1P receptors of mice infected with PR8 virus

**Figure 3:**
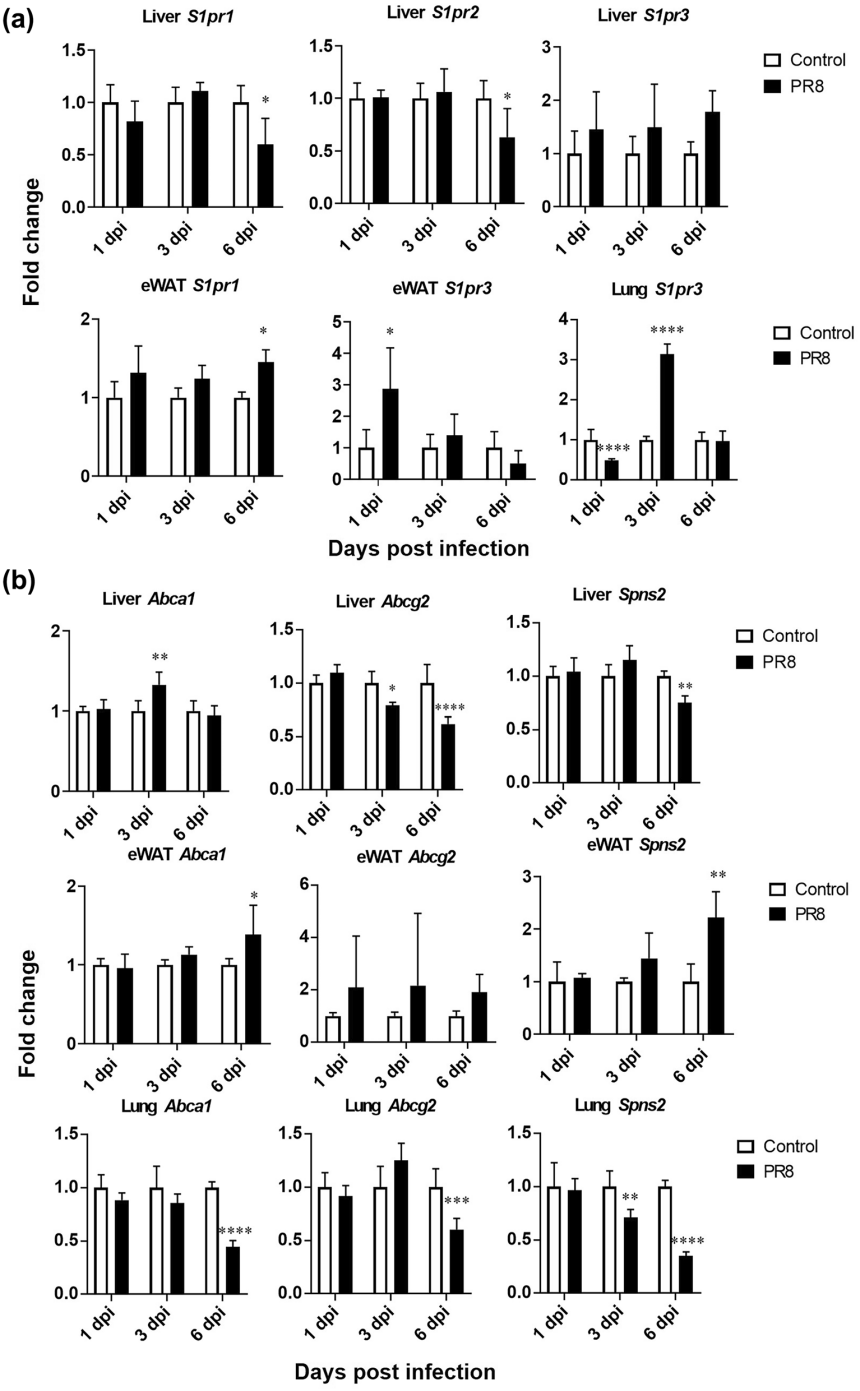
Expression levels of major S1P receptors (**a**) and transporters (**b**) in the liver, lung and e-WAT. Mice were intranasally inoculated with PBS alone or PBS comprising PR8 virus (500 PFU), and liver, e-WAT, lung, heart, and aorta samples were collected at 1, 3, and 6 dpi. Expressions of target genes normalized with that of 18S are presented as fold changes relative to those of the control mice at each time point. Bars represent means ± SEM of 7 or 8 (liver) or 3 or 4 (e-WAT) animals. White and black bars indicate data from control and PR8 virus-infected mice, respectively; *p < 0.05, 2way-ANOVA on ddCt, control vs. PR8 virus-infected mice at each time point.

S1P acts as an intracellular signaling molecule through five S1P-specific G-protein-coupled receptors termed S1PR1-5^20^. These receptors have overlapping or distinct expression patterns and functions in a cell- and tissue-type specific manner, resulting in various physiological functions of S1P in each organ^31^. The relative gene expressions of three major S1P receptors, *S1pr1-3* in the liver, lung, and e-WAT of mice infected with PR8 virus are shown in Fig. 3a. The gene expressions of *S1pr1* and *S1pr2* significantly decreased by twofold in the liver at 6 dpi, while there was no significant change in *S1pr3* levels (Supplementary Fig. S2). Although lung showed a decrease in the expression levels of *S1pr3* at 1 dpi, a significant increase up to threefolds was observed at 3 dpi. But the expression levels of *S1pr1* and *S1pr2* are unaltered in the lung*.* In contrast to the liver, e-WAT showed a slight increase in the levels of *S1pr1* at 6 dpi and *S1pr3* at 1 dpi, although *S1pr2* levels did not change throughout.

### PR8 virus infection alters gene expression of S1P transporters in liver, lung, and e-WAT in mice

S1P is produced inside the cell and exported via transporters, such as ATP-binding cassette (ABC) transporters (*Abca1*, *Abcg2*, *Abcc1*) and sphingolipid transporter 2 (*Spns2*), to stimulate *S1prs* on the cell membrane in a paracrine and/or autocrine manner. Therefore, expression levels of S1P transporters indirectly regulate S1P-S1PR signaling. The gene expression levels of *Abca1*, *Abcg2*, and *Spns2,* in the liver, lung, and e-WAT are shown in Fig. 3b. In the liver, *Abca1* increased slightly at 3 dpi; however, no significant changes were observed at 1 and 6 dpi. On the other hand, *Abcg2* and *Spns2* decreased significantly by almost twofold at 6 dpi. Conversely, significant increases in *Abca1* and *Spns2* levels were observed in e-WAT at 6 dpi in the infected mice, compared to controls. Surprisingly, in the lung, all the three transporters, *Abca1*, *Abcg2*, and *Spns2* showed a significant decrease at 6 dpi. By the way, another ABC transporter *Abcc1* gene expression was not affected in liver, lung, and e-WAT tissues by the virus infection (Supplementary Fig. S1). These results may suggest that the extracellular trafficking of S1P was reduced in the liver and enhanced in the e-WAT.

### Alteration of complex sphingolipids in the liver, lung, and plasma of mice infected with PR8 virus

**Figure 4:**
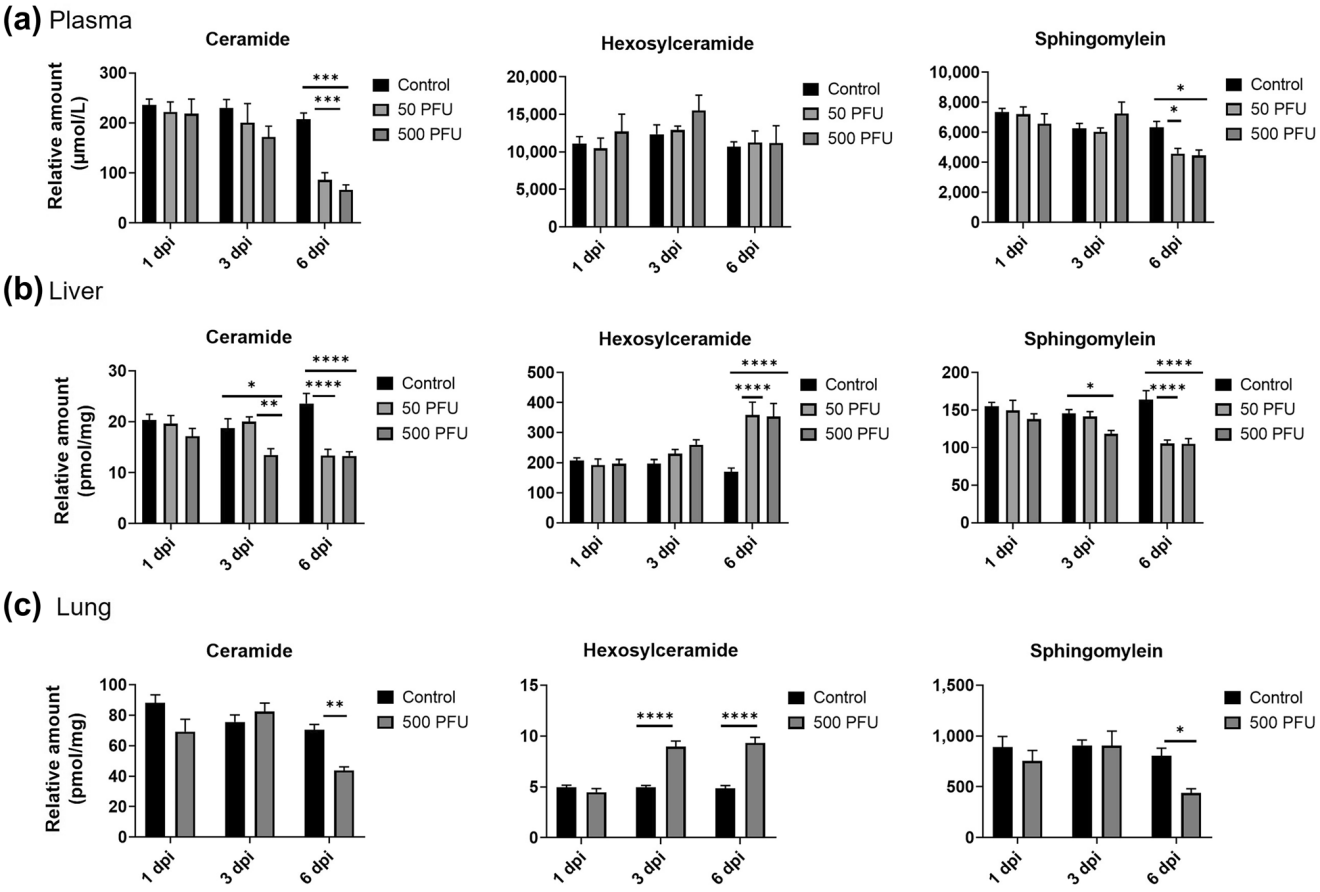
Relative concentrations of total ceramide, hexosylceramide and sphingomyelin in plasma (**a**), liver (**b**), and lung (**c**) of PR8 virus infected mice (n = 5 each, mean ± SE, 2way ANOVA with Tukey’s multiple comparison test, *p < 0.05, **p < 0.005, ***p < 0.0005).

The concentrations of other complex sphingolipids of the S1P pathway such as ceramides, hexosylceramide, and sphingomyelin in the liver, lung, and plasma of mice infected with PR8 virus were determined by untargeted LC/MS analysis (Fig. 4). A significant reduction in total ceramide and sphingomyelin levels was detected in the liver, lung, and plasma, particularly at 3 or 6 dpi, respectively. In contrast, the hexosylceramide levels were increased in the liver and lung but unchanged in plasma. These results demonstrate a significant influence of influenza virus infection on sphingolipid metabolism in plasma, liver, and the lung.

## Discussion

Viral infection triggers a specific lipid mediator of the host which, could serve as a potential biomarker to assist diagnostics^32^, and targeting host-derived lipid mediators appears to be a promising strategy to develop novel antiviral therapeutics^33^. The significance of an S1P/S1PR1 axis in virus infection has been previously reported^27^. However, there is little information on the kinetic profile of sphingolipids and S1P signaling in tissues other than the lungs during influenza. Here we investigated the changes of the levels of S1P and its related gene expressions upon PR8 virus infection in mice as summarized in Table 1.

**Table 1:** Summary of altered S1P levels and its pathway related gene expressions (Sph: sphingosine).

	Plasma	Liver	Lung	Aorta	Heart	e-WAT
Lipid metabolites	Sph (→) S1P (↑)	Sph (↑) S1P (→)		–	–	–
S1P related enzymes	–	*Sphk1* (↑) *Sgpl1* (↓)	*Sphk1* (↑) *Sgpl1* (↑)	*Sphk1* (↑)	*Sphk1* (↑)	*Sphk1* (↑) *Sgpl1* (↓)
S1P receptors	–	*S1pr1* (↓) *S1pr2* (↓)	*S1pr3* (↑)	*S1pr1* (↓) *S1pr2* (↓)	–	*S1pr1* (↑) *S1pr3* (↑)
S1P transporters	–	*Abca1 *(↑) *Abcg2* (↓) *Spns2* (↓)	*Abca1* (↓) *Abcg2* (↓) *Spns2* (↓)	–	–	*Abca1 *(↑) *Spns2* (↑)

The present results demonstrate the elevation of plasma S1P in mice with severe influenza (PR8 virus of 500PFU) on 6 dpi when the animals showed almost 25% of their body weight loss, dehydration, and decreased motility. Interestingly, 12-h-fasting has been reported to cause 5.5% body weight loss and to elevate S1P level in the circulation via increased lipid flux in mice^34^. Therefore, influenza-associated anorexia and consequent body weight decrease due to virus infection may have promoted lipid release from tissues and contributed to the elevation of S1P. However, given that the plasma sphingosine levels were not altered in mice infected with 50PFU and 500PFU PR8 virus for 6 and 3 days, respectively, when the animals showed around 15% weight loss, it is unlikely that S1P levels elevated simply due to increased lipid flow. Therefore, our data clearly indicate an impact of virus infection on S1P signaling rather than fasting-associated lipid release alone. In addition, since S1P is abundantly contained in red blood cells, it is possible that the elevated plasma S1P levels observed in the infected mice were caused by hemolysis. To exclude this possibility, plasma hemoglobin levels were measured, and there was no significant difference in hemoglobin levels between control and infected mice at any time points (Supplementary Fig. S3). Therefore, the increase in plasma S1P levels in infected mice is considered to be associated with changes in S1P metabolism as described below.

The increased gene expression of *Sphk1*, which mediates the conversion of S1P from its precursor sphingosine, was observed in the liver, lung, e-WAT, heart, and aorta of infected mice in this study. This observation is consistent with those in previous studies representing the induction and phosphorylation-dependent activation of SPHK1 by influenza virus infection or inflammatory cytokines in cultured cells (described later in detail)^26,35,36^. The induction of SPHK1 may result in an increase of S1P synthesis in tissues, which can explain the elevation of blood S1P levels in infected mice in this study and another previous one^37^. However, it is unclear what tissues and/or cells actually contributed to the elevation of plasma S1P at this moment. Future studies will be needed, and the possibility of increased release of S1P from red blood cells, which are the most important source of S1P in the blood^22^, should also be examined.

In contrast to the plasma, the liver and lung of infected mice showed increased concentration of sphingosine and unchanged S1P on 6 dpi. In this study, we focused on the regulation of *Sphk1/2* gene expression, which has been shown to be affected by a viral infection in detail, in order to investigate S1P metabolism during influenza. However, the concentration of sphingosine, a precursor of S1P, was rather increased in the liver of infected mice that showed increased *Sphk1* expression. When combined with the fact that ceramide levels in the liver and lung were decreased upon the infection, it seems that not only the production of S1P but also the entire metabolism of sphingolipids is affected in the tissue by influenza virus infection in a complex manner.

In addition to the liver, gene expression levels of enzymes involved in S1P metabolism were found to be significantly altered by the infection in the lung. The increase in lung *Sgpl1* gene expression observed in this study may suggest the reduction of S1P in the lung. The level of S1P in the lungs tended to decrease at 6 dpi, although the change was not statistically significant. In the endotoxin-induced lung injury model, the induction of *Sgpl1* contributes significantly to the decrease in lung S1P concentration induced by endotoxin administration, and the inhibition of SGPL1 activity alleviates the degree of lung injury^38^. Therefore, the increase in *Sgpl1* gene expression observed in this study may also be related to lung injury in influenza. Investigating the effects of viral infection on the expression and activities of S1P and other sphingolipid-metabolizing enzymes may enhance our understanding of the host response to infection.

The S1P signaling has already been demonstrated to be affected by various infectious diseases through the alteration of expressions or activities of related enzymes, particularly SPHK1. Interestingly, the effects of virus infection on the regulation of S1P are different among viruses. The increased gene expression and enzymatic activity of SPHK1 upon infection with influenza virus, respiratory syncytial (RS) virus, and human cytomegalovirus (HCMV) have been reported in cultured cell systems^26,35,39,40^. On the other hand, dengue virus type-2 infection reduces SPHK1 activity through a post-translational mechanism in multiple cell lines^41^. Considering changes in gene expressions of *S1pr*s and transporters observed in each tissue in this study, S1P signaling was expected to be down-regulated in the liver and up-regulated in the e-WAT in mice with severe influenza. Given the anti-inflammatory function of S1P, we speculate that increased plasma S1P levels can be protective against the infection as demonstrated in previous studies that S1PR1 agonists protected the host from influenza-virus-infection-induced acute immunopathological damages by suppressing inflammatory signaling^11,27^. However, since activated S1P signaling induces insulin resistance in adipose tissue by promoting inflammation^42^, the expected changes of S1P signaling in the e-WAT may be associated with energy metabolism disorders in severe influenza reported so far^1,7,8^. It is also interesting to note that the effect of influenza virus infection on changes in gene expression levels related to sphingolipid metabolism differs among tissues.

In addition to the increase in plasma S1P, the decreased levels of ceramide and sphingomyelin in the liver, lung, and plasma or increased level of hexosylceramide in the liver and lung further suggest the alteration of the sphingolipid metabolic pathways during severe influenza. Given the divergent roles of sphingolipids, particularly ceramide in viral infections from the suppression of viral replication to the enhancement of immune responses^43,44^, further investigation will be needed to elucidate the detailed mechanisms and roles of S1P and other sphingolipids in different tissues during influenza and other infectious diseases.

In summary, the present results indicate that influenza virus infection induces *Sphk1* gene expression in multiple tissues to elevate the level of circulating S1P at the lethal phase of severe influenza. Understanding the relationship between the influenza virus infection, S1P metabolism, and its signal transduction would provide novel insights into the pathogenesis of infectious diseases and host responses.

## Supplementary Information

Supplementary Information.

Supplementary Table S1.

## Data Availability

The datasets obtained during the current study are available from the corresponding author on a reasonable request.
