# Supplementary material for: Defining the kinetic effects of infection with influenza virus A/PR8/34 (H1N1) on sphingosine-1-phosphate signaling in mice by targeted LC/MS
Source: Sci Rep. 2021 Oct 11;11:20161. doi: 10.1038/s41598-021-99765-0 (PMC8505484; doi:10.1038/s41598-021-99765-0)
Supplement: Supplementary file 1 — Supplementary Information. [file 41598_2021_99765_MOESM1_ESM.docx]

Supporting information for

**Defining the kinetic effects of infection with influenza virus A/PR8/34 (H1N1) on sphingosine-1-phosphate signaling in mice by targeted LC/MS**

Divyavani Gowda^1#^, Marumi Ohno^3#^, Siddabasave Gowda B. Gowda^1^, Hitoshi Chiba^1,2^, Masashi Shingai^3^, Hiroshi Kida^3^*, and Shu-Ping Hui^1^*

1. Faculty of Health Sciences, Hokkaido University, Kita-12 Nishi-5, Kita-Ku, Sapporo 060-0812, Japan
2. Department of Nutrition, Sapporo University of Health Sciences, Nakanuma Nishi-4-3-1-15, Higashi-Ku, Sapporo 007-0894, Japan
3. International Institute for Zoonosis Control, Hokkaido University, Kita 20 Nishi10, Kita-ku, Sapporo, 001-0020, Japan

# Equally contributing authors

***Correspondence:**

1. Prof. Shu-Ping Hui

Faculty of Health Sciences, Hokkaido University, Kita-12 Nishi-5, Kita-Ku, Sapporo 060-0812, Japan ORCID: 0000-0001-9973-6461.

e-mail address: [keino@hs.hokudai.ac.jp](mailto:keino@hs.hokudai.ac.jp)

2. Prof. Hiroshi Kida

International Institute for Zoonosis Control, Hokkaido University, Kita 20 Nishi10, Kita-ku, Sapporo, 001-0020, Japan.

e-mail address: kida@vetmed.hokudai.ac.jp.

**Supplementary Figure S1**: Typical extracted ion chromatograms of sphingosine and S1P

**
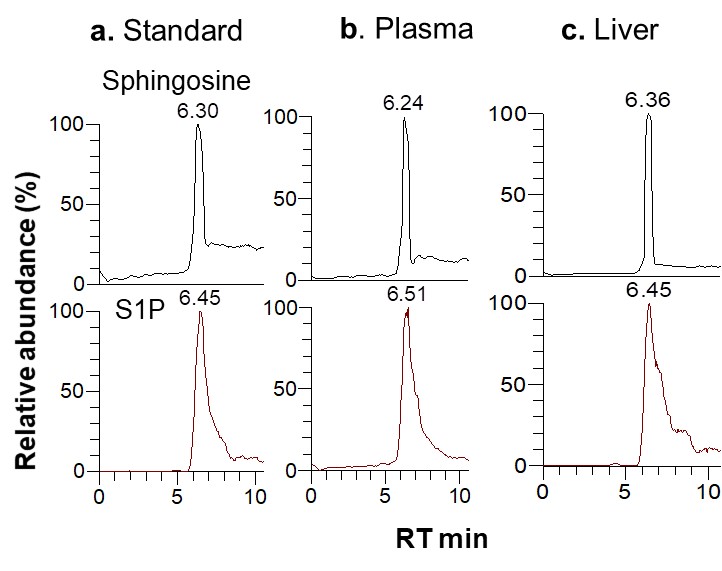
**

**Supplementary Figure S2**:

Mice were intranasally inoculated with PBS alone or PBS comprising PR8 virus (500 PFU), and liver, lung, e-WAT, heart, and aorta samples were collected at 1, 3, and 6 dpi. Expressions of target genes normalized with that of 18S are presented as fold changes relative to those of the control mice at each time point. Bars represent means ± SEM of 7 or 8 (liver, lung, aorta) or 3 or 4 (e-WAT, heart) animals. White and black bars indicate data from control and PR8 virus-infected mice, respectively; **p* < 0.05, ***p* < 0.005, ****p* < 0.0005, 2way-ANOVA on ddCt, control vs. PR8 virus-infected mice at each time point.


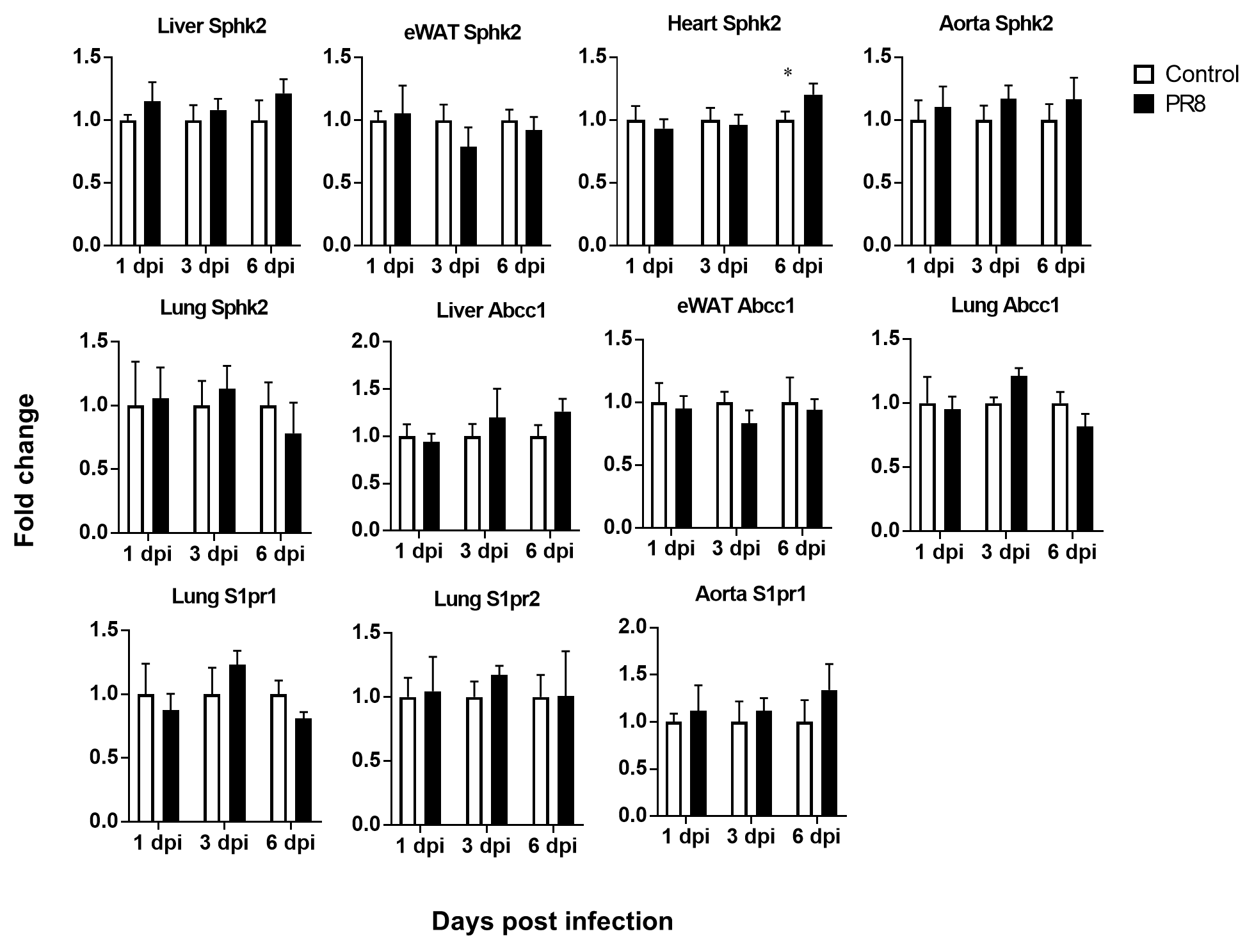


**Supplementary Figure S3**:

Mice were intranasally inoculated with PBS alone or PBS comprising PR8 virus (500 PFU), and plasma samples were collected at 1, 3, and 6 dpi. The hemoglobin levels in 10 µL of plasma were determined using a Hemoglobin Colorimetric Detection Kit (EIAHGBC, Thermo Fisher Scientific) according to the manufacturer’s protocol. White and gray bars represent means ± SEM of 5 control and infected animals, respectively. Statistical analysis was performed with 2way-ANOVA with Sidak’s multiple comparison test (NS: not significant).

**Untargeted analysis of sphingolipids using Linear Trap Quadrupole (LTQ)-Orbitrap MS:**

About 100 µl (10 mg) of the liver homogenate or 50 µl of plasma were transferred to an Eppendorf tube, followed by addition of 100 µL of the internal standard mixture in methanol (13.5 µM of sphingomyelin (d18:1/18:0(d9)) and 18.8 µM of ceramide (d18:1/15:0 (d7)) (Avanthi Polar lipids, Alabaster AL, USA) and the mixture was vortexed at 3500 rpm for 30 s. Subsequently, 400 µL of chloroform were added and vortexed for 5 min, and 100 µL of milli-Q were added with an additional vortex for approximately 30 s. The biphasic extracts were centrifuged at 15000 rpm for 10 min at 4°C, the lower chloroform layer was transferred to a vial, while the upper layer was re-extracted with an additional 400 µL of chloroform. The chloroform extracts were combined and concentrated under vacuum. The dried total lipids were redissolved in 100 µL of methanol with gentle vortexing and centrifuged. Approximately 10 µL of each sample was injected into the LC/MS via an autosampler.

The lipidomic analysis was performed using a prominence UHPLC system (Shimadzu Corp., Kyoto, Japan) coupled with an LTQ Orbitrap MS (Thermo-Fisher Scientific Inc., San Jose, CA) and an Atlantic T3 C18 column (2.1 × 150 mm, 3 µm, Waters, Milford, MA) at 40 °C. The flow rate of the mobile-phase (A: aqueous 10 mM CH_3_COONH_4_, B: isopropanol, C: methanol) was set to 200 μL/min with a linear flow of: 30% B and 35% C (0-1 min), 80% B and 10% C (1-14 min), 85% B and 10% C (14-27 min) in negative mode. In brief, MS data were acquired in electron spray ionisation (ESI)-negative mode with the capillary temperature: 330 °C, sheath gas flow: 50 units, and auxiliary gas: 20 units. The source voltage was set to 3 kV, and the capillary voltage was set to -10 V, A Fourier transform (FT) full scan range was set to *m/z* 160–1900 to acquire MS^1^ spectra for high-resolution masses. Low-resolution MS/MS spectra were obtained at a collision energy of 40 V in ion-trap mode. The raw data were processed using MS DIAL (version 4.2) software for the alignment and identification of lipid species Further, each identified lipid species was confirmed by MS/MS spectral matching with the built-in reference library of MS DIAL. The quantification of lipid molecules was carried out according to the definition of Lipidomics Standards Initiative level 2 and level 3, which was achieved if the lipid molecule was quantified by the labelled internal standard of the same lipid subclasses or representative lipid class category. The data were normalised by the weight or volume of the sample, and the concentration (in pmol/mg or µmol/L) of the lipid molecular species was calculated by taking the peak intensity ratios of the analyte to the internal standard and multiplying it by the pmol of the added internal standard.
